# A mixed-methods program evaluation of the Alda Healthcare Experience- a program to improve healthcare team communication

**DOI:** 10.1186/s12909-022-03972-w

**Published:** 2022-12-28

**Authors:** Heid Preis, Mallory Dobias, Katherine Cohen, Elizabeth Bojsza, Clare Whitney, Susmita Pati

**Affiliations:** 1 Department of Psychology, Stony Brook University, Stony Brook, NY USA; 2 Department of Obstetrics, Gynecology, and Reproductive Medicine, Renaissance School of Medicine, Stony Brook University, Stony Brook, NY USA; 3 Alda Center for Communicating Science, Stony Brook University, Stony Brook, NY USA; 4 School of Communication and Journalism, Stony Brook University, Stony Brook, NY USA; 5 School of Nursing, Stony Brook University, Stony Brook, NY USA; 6 Center for Medical Humanities, Compassionate Care and Bioethics, situated in the Department of Family, Population and Preventive Medicine, Renaissance School of Medicine, Stony Brook University, Stony Brook, NY USA; 7 Department of Pediatrics, Renaissance School of Medicine, Stony Brook University, Stony Brook, NY USA

**Keywords:** Medical improvisation, Alda healthcare experience, Interprofessional healthcare teams, Communication training, Healthcare workers burnout, A mixed-methods program evaluation of the Alda Healthcare Experience- a program to improve healthcare team communication

## Abstract

**Background:**

Communication among interprofessional healthcare worker teams is critical to ensure a thriving and resilient workforce. We will evaluate the implementation and effectiveness of the Alda Healthcare Experience (AHE), a novel medical improvisation (improv) workshop designed to improve interprofessional communication skills among healthcare professionals. The AHE workshop includes a two-hour experiential training workshop led by an improv specialist and a clinical co-facilitator. In July 2022 we began implementing the AHE workshop by training 18 clinical co-facilitators who will co-facilitate the workshops for 550 healthcare workers from five hospital departments at Stony Brook University Hospital over the course of a year and a half. Using mixed-methods, we will conduct an Effectiveness-Implementation Hybrid Design project that includes an outcome evaluation (effectiveness) and a process evaluation (implementation).

**Methods:**

Our outcome evaluation will assess the impact of the AHE workshop on short- and long-term improvement in interprofessional communication, stress, and professional fulfillment. The process evaluation component will examine programmatic, organizational, and individual facilitators or barriers to effective implementation of the AHE workshop. Qualitative methods will include dimensional analysis employing individual interviews of 20–40 AHE Project Participants, 5–10 Selected Informants, and all the clinical co-facilitators. Quantitative methods will use a quasi-experimental longitudinal design with an intervention group and surveillance of a control group (wait-list) and repeated assessments using validated instruments measuring communications skills, professional fulfillment, stress, burnout, uncertainty tolerance, and teamwork.

**Discussion:**

Effective and efficient communication within healthcare teams is fundamental to building team cohesion that, in turn, supports individual resilience and builds positive organizational culture. The AHE program is an innovative approach to improve interprofessional healthcare communication and reduce healthcare worker burnout. In addition to institutional buy-in, rigorous evaluations of medical improv programs are necessary as a critical step in making such programs scalable.

**Trial registration:**

N/A

## Background

Healthcare worker burnout is a major public health concern that can negatively impact both the well-being of healthcare workers as well as the patients and communities they serve [1–3]. Burnout is a result of many interconnected factors; one key factor includes poor interprofessional communication [4]. Interprofessional communication is multifaceted, including communication across different medical specialties (e.g., surgery and anesthesiology), different professional specialties (e.g., physicians and nurses), and different levels of training (e.g., attendings and trainees [5]). When interprofessional communication is minimal or burdensome, clinicians are likely to face increased mental and physical workload, which may cause significant distress and burnout. Furthermore, miscommunication is a major cause of medical errors [6, 7], leading to loss of life and to economic burden. Effective team communication among healthcare workers is critical to ensure a thriving resilient workforce and support equitable, culturally competent, and effective healthcare delivery [8–13].

The Alda Healthcare Experience (AHE) communication workshop is a brief, evidence-based medical improvisation (improv) communication workshop that could be beneficial for healthcare workers. The primary goal of the AHE workshop is to improve provider resilience by improving communication skills among individuals and within healthcare teams, increase individuals’ ability to tolerate uncertainty, enhance team cohesion, improve organizational culture, and subsequently reduce burnout and stress and improve professional fulfillment and patient outcomes. Medical improv, which is an application of theater arts improv, is emerging as a promising approach in medical education including among health care students [14–18]. The improv process requires active listening skills and continued connection and collaboration with others. It requires participants to empathetically anticipate the needs of others [16, 19, 20]. Such skills are critical for effective communication among interprofessional healthcare teams [21, 22]. In the last few years, several studies have suggested that medical improv can improve communication between healthcare workers and patients [17, 23, 24].

The AHE’s innovative features are that it is short (2 hours), team-taught, and grounded in social science theory. The AHE includes specific experiential and reflective activities designed to prompt real-world application of team communication skills in healthcare settings, and draws upon the theater arts and the discipline of science communication [16, 25–27]. Preliminary evidence piloting the AHE workshop in October 2020 among healthcare professionals from the Anesthesiology Department at Stony Brook University, indicate that the AHE program is feasible, acceptable, engaging, and effective in generating expected participation outputs and achieving its immediate outcomes [28, 29]. However, rigorous evaluations of medical improv programs for interprofessional healthcare workers have not been published in the peer-reviewed literature. Evaluating programmatic aspects of such medical improv workshops (process evaluation) and of its impacts (outcome evaluation) are necessary critical steps in making such programs scalable.

## Methods

### Project aims & design

Grounded in implementation science and program evaluation [30], the present project will evaluate the implementation and effectiveness of the AHE, a medical improv workshop designed to improve communication skills among healthcare professionals. Specifically, using mixed methods, we will conduct an Effectiveness-Implementation Hybrid Design project [31] that includes [1] an outcome evaluation to determine the impact of this workshop on communication skills among healthcare professionals (effectiveness), and [2] a process evaluation to explore interprofessional communication and its effects on AHE implementation fidelity (implementation).

**Fig. 1:**
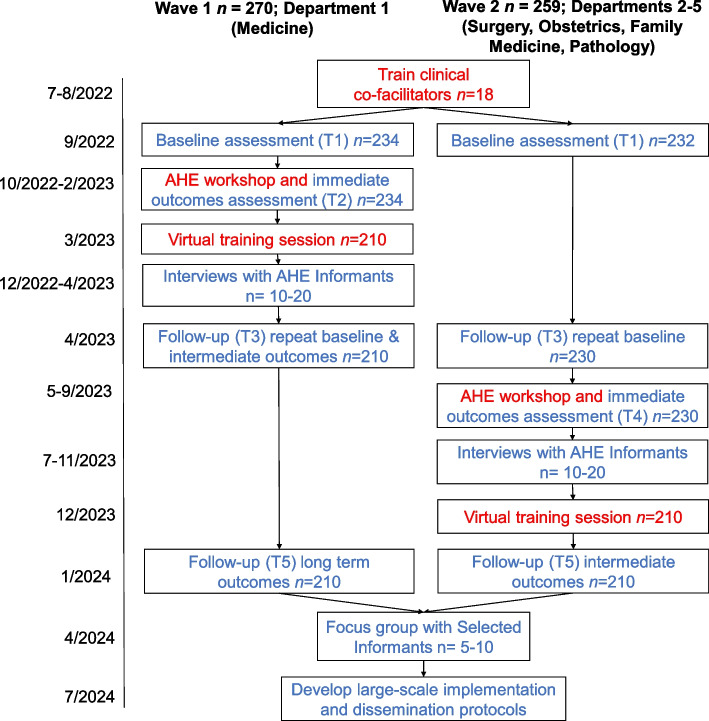
Evaluation design

Our outcome evaluation will assess the impact of the AHE workshop on short- and long-term improvement in several relevant outcomes, including interprofessional communication, stress, and professional fulfillment. We will use an embedded experimental explanatory sequential mixed methods design for implementation and evaluation [32, 33]. The evaluation will use participatory action research as a guiding framework and will include both qualitative methods (e.g., dimensional analysis employing individual interviews) and quantitative methods (e.g., quasi-experimental longitudinal [34–36]). In the first phase (see Fig. 1), we will deliver the AHE workshops and conduct a quantitative prospective outcome evaluation to assess the intervention effects using a quasi-experimental approach with an intervention group and surveillance of a control group (wait-list).

Next, our mixed-methods process evaluation will examine programmatic and individual barriers or facilitators to effective implementation of the AHE medical improv communication workshop. This evaluation will consist of qualitative interviews and quantitative analyses to explore organizational-, departmental-, and individual-level factors, related to interprofessional communication and its effects on delivery, fidelity, and effectiveness of the intervention. We will use our quantitative findings to inform the selection of AHE Informants (key AHE Stakeholders and AHE Project Participants) for qualitative in-depth interviews focused on interprofessional communication and the AHE workshop experience. In addition, we will conduct repeated brief interviews with clinical co-facilitators after AHE workshop sessions. Qualitative interviews will be aligned with dimensional analysis method [37, 38].

### Alda healthcare experience intervention

The AHE workshops will target approximately 550 healthcare professionals and nurses—both practicing professionals and trainees. Physicians, resident physicians and fellows, and practicing nurses will be drawn from five Stony Brook University Hospital Departments (Surgery, Medicine, Obstetrics/Gynecology, Family Medicine, and Pathology) whose leaders have indicated their support for this project. Participation in the AHE workshop will be mandatory and free; participants will also receive continuing education credits (e.g., Continuing Medical Education, Continuing Nursing Education) for their attendance. All personnel trained in the AHE workshop will be eligible to participate in the program evaluation.

As suggested in the literature [14], each workshop is co-facilitated by two leaders, an improv-trained facilitator and a clinically trained facilitator. Using a train-the trainer approach, clinical professionals will be nominated by leadership from each participating department to receive specialized training by the Alda Center team to enable them to co-facilitate the workshops and be ambassadors of change in their department. Approximately 18 clinical co-facilitators will be trained by going through 2 3-hour group sessions followed by 1-hour (maximum) 1:1 coaching. The clinical co-facilitator training includes experiencing the AHE workshop as a participant (novel to the AHE). Participation in the evaluation component, in addition to the training, will be voluntary.

The Alda Center for Communicating ScienceⓇ has assigned three improvisation facilitators to this project. All three have extensive experience designing and facilitating experiential learning using the Alda MethodⓇ, and have taught prior versions of this curriculum. In addition to facilitating the workshops, one facilitator will serve as the designated liaison to the project team, lead curriculum designer and lead clinical co-facilitator trainer.

Based on the fundamentals of medical improv and experiential learning, the AHE program includes a two-hour in-person workshop comprised of a series of improvisational exercises that focus on specific communication skills (recognizing the give and take of relational communication, listening to collaborate, reflecting values that are shown through passion, thinking creatively and generativity about circumstances) followed by a discussion about practical application in healthcare [14–16]. The exercises are immersive and participatory - participants work in pairs or groups to complete each exercise, and then engage in a full group discussion guided by open-ended questions from the facilitators. At key points in the workshop, participants are given written prompts to reflect. Follow-up one-hour virtual workshops will be conducted 1–3 months after the workshop in groups of up to 30 aimed to refresh the principles of improvisation to the workshop participants and introduce to a new tool to help them strategize a process to start challenging interprofessional conversations with colleagues.

Based on pilot data, to ensure maximum participation, groups will include 7–10 healthcare professionals, an improv facilitator, and a clinical co-facilitator. There will be approximately 68 separate AHE in-person workshops with a total of *n* = 100 healthcare professionals trained by the end of 2022, *n* = 350 more in 2023, and *n* = 100 more in the first quarter of 2024.

### Participants

Three types of participants will be involved in the present evaluation: AHE participants and stakeholders, clinical co-facilitators, and improv facilitators (see Table 1). AHE participants who choose to participate in the evaluation will be asked to join the project and complete online questionnaires. AHE Project Participants will receive $5 gift cards for each completed questionnaire. We expect 85% of the healthcare professionals that take part in the AHE workshop to agree to become AHE Project Participants for a baseline of *N* = 468 (*n* = 234 in each group). In addition, 20–40 AHE Project Participants and AHE Stakeholders will be chosen to be AHE Informants and take part in qualitative interviews. Of the AHE Informants, 5–10 will be chosen as Selected Informants and participate in a subsequent focus group. AHE Informants will be purposefully sampled based on individual characteristics and informed by the quantitative findings. AHE Informants will be chosen to reflect different professions, career levels, departments, racial and ethnic backgrounds, and gender identities. They will receive a $15 gift card for their time. As part of their role, all clinical co-facilitators will be interviewed following each AHE workshop they lead. With their agreement, these interviews will be used in real-time to improve the facilitation process as well as be used for evaluation purposes. Each co-facilitator will be eligible to receive a $3000 stipend for their roles (e.g., training, co-facilitating 4–6 AHE workshops, interviews). Improv facilitators will complete a brief online questionnaire following each AHE workshop session they lead. Their hourly pay is commensurate with experience.

**Table 1:** Project participants

Participant	Role in project	***N***	Project procedure	Payment
AHE Project Participants	AHE workshop participants who agree to participate in evaluation	468	Four brief online questionnaires	$5/ survey
AHE Informants	AHE Project Participants who agree to be interviewed and AHE stakeholders	20–40	40–60 minute individual interview	$15/ interview
Selected Informants	Chosen from AHE Informants	5–10	Focus group interviews	$15/ group
Clinical co-facilitators	Get training and co-lead 4–6 AHE workshops	18	10–30 minute individual interviews after each AHE workshop they co-lead	$3000 Stipend (max)
Improv facilitators	Lead 15–30 AHE workshops	3	Brief online questionnaire after each AHE workshop they lead	Hourly

The project was reviewed by the Stony Brook University IRB (IRB2022–00231) and approved as a project that is not considered human subject research based on Common Rule determination. Nonetheless, prior to completing the baseline survey, AHE workshop participants were presented with a brief description of the evaluation project, their involvement, confidentiality, and compensation. Participants who were interviewed were presented similar information about their involvement in the evaluation verbally as well.

### Procedures- quantitative

For the AHE Project Participants, evaluation procedures include online surveys collected during key time points for the AHE (see Fig. 1). AHE participants who are interested, will be asked to become AHE Project Participants and complete four online surveys over the course of 2 years (see Table 2) that will be administered through Qualtrics. Surveys will take approximately 3–5 minutes to complete and will repeatedly assess constructs related to teamwork, burnout, stress, well-being, and communication. The following previously validated measures will be included in the surveys:

**Table 2:** AME Project Participants quantitative measures

Construct	Measure	# items	Time points
Communication Skills	Perceived Interpersonal Communications Skills Scale (PICSS) [28]	8	1, 2, 3, 4, 5
Cohesion	Inclusion of Other in the Self Scale (IOS) [39]	1	1, 3, 5
Teamwork and Response to Errors	Surveys on Patient Safety Culture (SOPS) [40]	7	1, 3, 5
Uncertainty Tolerance and need for predictability	Uncertainty Tolerance Scale (UTS) [41, 42]	8	1, 5
Stress	Short Perceived Stress Scale (PSS-4) [43]	4	1, 3, 5
Work Exhaustion and Professional Fulfillment	Stanford Professional Fulfillment Index (PFI) [45]	9	1, 3, 5
Well-Being	The PROMIS Meaning and Purpose self-report scale [46]	4	1, 3, 5
Engagement in workshop	Visual Analog Scale	1	2 or 4

#### Communication skills

Communication will be assessed at all time points using the eight item Perceived Interpersonal Communications Skills Scale (PICSS) that was previously developed by the Alan Alda Center for Communicating Science [28]. Each item assesses a different skill related to flexible empathic communication (e.g., “Beginning a conversation with an open mind”). Participants are asked to rate their skill level for each communication item on a Likert-type scale ranging from 1= “not skilled at all” to 5 = “very skilled”. This scale has shown validity in previous pilot studies (Cronbach’s alpha is between 0.89 and 0.91; 28). The overall score is represented as the mean of responses. Higher scores indicate higher levels of communication skill.

#### Cohesion

Closeness with the healthcare team will be measured at time points 1, 3, and 5 using the Inclusion of Other in the Self Scale (IOS; 39). On a single item, respondents choose one of seven pairs of circles, labeled “Self” and “Other”, with differing amounts of overlap (1 = “no overlap” to 7 = “most overlap”). Higher scores indicate greater self-perceived cohesion between participants and others in their healthcare team.

#### Teamwork and response to errors

Teamwork and response to errors will be measured at time points 1, 3, and 5 using the Surveys on Patient Safety Culture questionnaire (SOPS; 40). Two subscales of the SOPS will be included in the current evaluation: three items measuring Teamwork (e.g., “In this unit, we work together as an effective team”) and four items measuring Response to Errors (“When an event is reported in this unit, it feels like the person is being written up, not the problem”). Participants are asked to respond to each statement on a Likert-type scale ranging from 1 = “strongly disagree” to 5 = “strongly agree”. The Cronbach’s alpha of the Teamwork subscale is 0.76. The Cronbach’s alpha of the Response to Errors subscale is 0.83. Higher scores indicate greater teamwork and effective responses to errors.

#### Uncertainty tolerance

The Uncertainty Tolerance Scale (UTS; 41,42) will be used to assess uncertainty tolerance at time points 1 and 5. The UTS includes two subscales: five items measuring Uncertainty Tolerance (Cronbach’s alpha is 0.70) and three items measuring Need for Predictability (Cronbach’s alpha is 0.73; 28). Respondents indicate the degree to which they agree or disagree with each of eight statements (e.g., “I like change and excitement”), on a scale ranging from 1 = “strongly disagree” to 6 = “strongly agree”. Higher scores indicate a greater need for predictability of a greater intolerance of uncertainty.

#### Stress

The Short Perceived Stress Scale (PSS-4; 43) will be used to assess self-reported stress at time 1, 3, and 5. Respondents will be asked to indicate how often each of four stated situations had occurred within the past month (e.g., In the last month, how often have you felt that you were unable to control the important things in your life?) on a Likert-type scale ranging from 0 = “never” to 4 = “very often”. Cronbach’s alpha for the PSS-4 is typically between 0.60 and 0.82 [44]. Higher scores indicate greater perceived stress.

#### Work exhaustion and professional fulfillment

Work exhaustion and professional fulfillment will be measured at time 1, 3, and 5 using the Stanford Professional Fulfillment Index (PFI; 45). We will use two subscales included in the PFI. The first subscale is Burnout which includes four items related to work exhaustion (e.g., “Physically exhausted at work”). The second subscale is Professional Fulfillment which includes five items related to satisfaction with work (e.g., “I feel happy at work”). Participants respond to each statement on a Likert-type scale ranging from 0= “not at all true” to 4 = “completely true” or 0= “not at all” to 4 = “extremely.” The Cronbach’s alpha of the Burnout subscale is 0.92 and the Cronbach’s alpha of the Professional Fulfillment subscale is 0.91 [45]. Higher scores on the Burnout subscale indicate greater work exhaustion. Higher scores on the Professional Fulfillment subscale indicate greater fulfillment.

#### Well-being

The PROMIS Meaning and Purpose self-report scale [46] will be used to measure well-being at time points 1, 3, and 5. Responses to each of four items (e.g., “My life has meaning”) are rated on a likert-type scale ranging from 1 = “strongly disagree” to 5 = “strongly agree”, or 1= “not at all” to 5 = “very much”). The Cronbach’s alpha for this scale is 0.90 [46]. Higher scores indicate greater wellbeing.

#### Excitement about AHE workshop

The extent to which AHE Project Participants are excited about participating in the workshop will be measured at time point 1 using a Visual Analogue Scale. Participants are asked to indicate “Using the scale, please indicate how excited you are about participating in the Alda Healthcare Experience communication workshop?” on a sliding scale from 0 = not excited at all to 100 = Extremely excited.

#### Engagement in workshop

The extent to which AHE Project Participants were actively engaged in the workshop will be measured at time 2 for Wave 1 and time 4 for Wave 2 using a Visual Analogue Scale. Participants are asked to indicate “How engaged you were in today’s workshop” on a sliding scale from 0 = not engaged to 100 = fully engaged.

#### Background data

AHE Project participants’ sociodemographic background (age, gender identification, race and ethnicity, degree/ credentials, and career level) will be assessed at time point 1. In addition, participants will be asked whether they have any prior experience with improv, and asked to indicate what kind of experience they have.

#### Employee engagement

Department-level employee engagement will be indexed by absenteeism (e.g., works days missed) and retention (e.g., turnover rates) every 6 months, starting with the 6 months before T1 and ending 6 months after the T5.

#### Debrief Improv facilitators surveys

Improv facilitators will complete a brief (2 minutes) online survey at the end of each AHE workshop session to document the workshop environment (e.g., distractibility, disruptions, perceived cohesion, initiation of questions, willingness to engage). Several outcomes (i.e., group size, disruptions, perceived cohesion, and engagement) will be collected, using original measures designed for this project.

### Procedures- qualitative

All qualitative data collection and management procedures will be carried out using institutionally secured and password protected video-conferencing and data storage software by a team of three to five project team members trained and supervised by the project qualitative lead co-investigator. Qualitative interviews will be conducted virtually by a project team member trained in qualitative data collection. Interviewers will use semi-structured interview and discussion guides to ensure data elicitation on topics central to the aims of the project, which will be iteratively refined in alignment with emerging concurrent analysis and theoretical sampling. Once completed and audio-recorded, each interview will be transcribed using an institutionally approved transcription software service, and at least two project team members will independently align audio-recordings with their corresponding transcripts to ensure verbatim accuracy and remove identifiable information.

For the qualitative process evaluation of the AHE, we will use theoretical sampling to select 20–40 individuals for in-depth individual interviews, from the pool of AHE Project Participants and AHE Stakeholders that agree to being contacted to participate in interviews. Should we face recruitment challenges resulting in fewer than expected interviews, theoretical sampling will enable sufficient depth of data for analysis through application of it during interviewing by “steering questions in the direction of emergent theorizing,” as well as after data collection is complete by theoretically sampling “within the data” [32 , p. 8]. An initial portion of the AHE Informants will be selected from the Wave 1 department to allow a critical mass for analysis (between T1-T3), then additional interviews will be conducted with Wave 2 AHE Project Participants and AHE Stakeholders (between T4-T5) to ensure sample heterogeneity in terms of clinical department, as well as overall data saturation. Each interview will last approximately 60 minutes and interview questions will focus specifically on participants’ experiences of interprofessional communication, burnout, and the AHE workshop.

For the qualitative stakeholder participatory validation (after T5), we will sample 5–10 Selected Informants from the AHE Informants to participate in a focus group interview. The focus group will last approximately 60 minutes and will aim to establish participatory validation of the theoretical findings generated from individual interviews as well as any proposed modifications to the AHE rooted in these findings or the quantitative outcome evaluation.

For the clinical co-facilitator analysis (between T1-T5), each clinical co-facilitator will participate in brief semi-structured interviews after every AHE workshop they facilitate. These individual interviews will last approximately 10–30 minutes and are designed to meet multiple project-related goals. First, the interviews will function as a practical opportunity to learn of implementation issues requiring troubleshooting prior to subsequent AHE workshop sessions, across all clinical and improv co-facilitators. Second, the interviews are guided by the underlying goal of understanding the longitudinal experiences of clinical co-facilitators involved in delivering improv-based communication workshops.

### Analysis plan- quantitative

Power analysis for the quantitative methods is based on our ability to conduct Structural Equation Modeling (SEM) among all participants and to detect differences between Wave 1 and Wave 2. For SEM we will need a final sample of *n* = 200, therefore, having a baseline sample of *n* = 234 AHE project participants (85% of all AHE participants in each Wave) in each Wave will ensure a sufficient final sample size of *n* = 210 in each wave (10% attrition over time). Having *n* = 234 in each Wave will also give us enough power to compare the two groups after controlling for individual and program level factors using techniques such as Hierarchical Linear Modeling.

Quantitative outcome analyses will examine inter-individual changes over time among AHE participants as well as departmental-level differences between intervention and control groups using independent and dependent t-tests. We will also examine sequential processes in outcomes using SEM. Our quantitative process evaluation will examine moderators for AHE impacts using PROCESS macros. Effects of individual (e.g., age, staff position), program (e.g., participants’ engagement, facilitator-rated group dynamics, time of day), and organizational (e.g., department, team structures) level factors on program outputs will be tested using nested techniques such as Hierarchical Linear Modeling. To reduce bias associated with attrition from the study over time, we will analyze missingness patterns and handle any missing data accordingly (e.g., pairwise deletion, multiple imputation).

### Analysis plan- qualitative

Inductive analysis of all qualitative data will be guided by dimensional analysis, an interactionist methodology [37, 38]. Each interview transcript will be coded over multiple passes by members of the project team trained in qualitative data analysis. First pass coding will involve a Gestalt read of the data and analytic memo writing. In subsequent passes, the data will be analyzed by generating open, axial, and theoretical codes. Constant comparison technique [47], dimensionalization [37, 38], and theoretical sampling [32] will guide each phase of iterative analysis, facilitated by regular dialogic engagement sessions to ensure research team coherence in analytic procedures.

Inductive analysis of the data collected for the qualitative process evaluation of the AHE will help identify theoretical relationships between key dimensions of healthcare team communication, safety issues, and attitudes towards communication training including workshop groups environment. For the qualitative stakeholder participatory validation, analysis of the data will seek to uncover dimensions relevant for program implementation – utility, feasibility, acceptability, and scalability. Analysis of clinical-co-facilitator interviews, will aim to identify theoretical relationships among concepts central to improv communication training among interprofessional healthcare workers.

## Discussion

This mixed-methods process and outcome program evaluation will examine the implementation and the effects of an innovative medical improv workshop. Over 550 healthcare professionals from five hospital departments will participate in the two-hour AHE workshops that will be led by an improv facilitator and a clinical co-facilitator followed by a 1-hour virtual booster session several months later. The robust Effectiveness- Implementation Hybrid Design project will help uncover the effects of the program, under what circumstances and for whom it works best, and what can be done to improve it. Project findings will inform the creation of large-scale implementation and dissemination protocols.

During the Coronavirus Disease 2019 (COVID-19) pandemic, the mental and physical burden placed upon healthcare workers was – and continues to be – heavy. There is evidence that clinicians experienced high rates of stress and burnout even before the pandemic began, suggesting that these concerns will not be fully resolved even if the number of COVID-19 cases is reduced [1–3]. However, the adoption of the AHE workshop is unique and innovative because it breaks away from traditional models to address burnout.

The successful implementation and rigorous evaluation of such a program requires institutional buy-in and sponsorship by organizational leaders. Leaders set the example by participating in the workshop as individuals, by providing ongoing collaboration to maximize participation across the organization, and by providing access to data required to rigorously evaluate the impact of the workshop. For this project, our institution’s Patient Experience Officer is serving as our executive sponsor, completed clinical co-facilitator training, and is facilitating our team’s access to organizational employee engagement data (i.e., rates of retention and absenteeism); furthermore, five department chairs enthusiastically agreed to have their staff participate and nominated co-facilitators to take a more active part in the program. The importance of having clinical co-facilitators lead medical improv workshops has been previously established [14]. From our experience, having the clinical co-facilitators from our institution and from the participating departments is as critical. This increases the authenticity of the workshops, helps the trainees be more at ease, and allows for application of the improv exercises to the institutional and departmental context. Moreover, these trained clinical co-facilitators will be ambassadors for change in communication culture within their department.

While the current project will address short-and long-term outcomes, sustainability of the communication impacts of the AHE workshop are limited to the one-year follow-up of the planned evaluation. In addition, the workshop is only delivered to five hospital departments and mainly delivered to practicing and training physicians and nurses and do not include ancillary health professionals (e.g., physical therapists, occupational therapists) who often have important roles in healthcare teams. While these five departments represent key departments within the organization and the healthcare professional make up the majority of the healthcare teams, it is unclear if this amount of reach will be enough to create sustainable cultural changes in our institution. One way to address this would be to make the AHE mandatory to all direct care staff and examine its institutional-level effects. However, this might be challenging in a highly matrixed environment where faculty are under different leadership than nursing, trainees, and ancillary staff.

Effective and efficient communication within healthcare teams is fundamental to building team cohesion that, in turn, supports individual resilience and builds positive organizational culture. Healthcare teams are very well trained to communicate well during patient “codes” (e.g., when a patient has a cardiopulmonary arrest requiring a team of healthcare providers to rush to the specific location and begin immediate resuscitation efforts). In contrast, most healthcare professionals graduate from their respective professional schools with little training in the interprofessional team communication skills that are needed to provide high quality and equitable healthcare to patients. Moreover, the majority of current performance expectations and career advancement pathways for healthcare professionals– especially those in academic medical centers– fail to reward collaboration. There have been dramatic changes in the workload and workflow for healthcare professionals over the past 20 years. Healthcare policymakers and organizations now need to invest in the needs of the workforce to ensure a robust and resilient future for us all.

## Data Availability

Not applicable.

## Abbreviations

AHE: Alda Healthcare Experience
COVID-19: Coronavirus Disease 2019
SEM: Structural Equation Modeling

## References

[1] Dyrbye LN, West CP, Hunderfund AL, Sinsky CA, Trockel M, Tutty M (2020). Relationship between burnout, professional behaviors, and cost-conscious attitudes among US physicians. J Gen Intern Med.

[2] Dyrbye LN, Shanafelt TD, Johnson PO, Johnson LA, Satele D, West CP (2019). A cross-sectional study exploring the relationship between burnout, absenteeism, and job performance among American nurses. BMC Nurs.

[3] Geiger-Brown J, Lipscomb J (2010). The health care work environment and adverse health and safety consequences for nurses. Annu Rev Nurs Res.

[4] Vermeir P, Blot S, Degroote S, Vandijck D, Mariman A, Vanacker T (2018). Communication satisfaction and job satisfaction among critical care nurses and their impact on burnout and intention to leave: a questionnaire study. Intensive Crit Care Nurs.

[5] Real K, Buckner MM (2014). Interprofessional communication: health care teams and medical Interpreters1. Health Communication.

[6] Joint Commission. Sentinel Event Data Root Causes by Event Type: 2004–2015 [Internet]. 2017. Available from: https://hcupdate.files.wordpress.com/2016/02/2016-02-se-root-causes-by-event-type-2004-2015.pdf.

[7] Sutcliffe KM, Lewton E, Rosenthal MM (2004). Communication failures: an insidious contributor to medical mishaps. Acad Med.

[8] Medicine I of. Crossing the Quality Chasm: A New Health System for the 21st Century. 2001 [cited 2022 Sep 30]. Available from: https://nap.nationalacademies.org/catalog/10027/crossing-the-quality-chasm-a-new-health-system-for-the.

[9] Medicine I of. The 1st Annual Crossing the Quality Chasm Summit: A Focus on Communities: Report of a Summit. 2004 [cited 2022 Sep 30]. Available from: https://nap.nationalacademies.org/catalog/11085/the-1st-annual-crossing-the-quality-chasm-summit-a-focus. 25009886

[10] Dzau VJ, Kirch DG, Nasca TJ (2018). To care is human — collectively confronting the clinician-burnout crisis. N Engl J Med.

[11] Kreitzer MJ, Klatt M (2017). Educational innovations to foster resilience in the health professions. Med Teach.

[12] Lemieux-Charles L, McGuire WL (2006). What do we know about health care team effectiveness? A review of the literature. Med Care Res Rev.

[13] Rosen MA, DiazGranados D, Dietz AS, Benishek LE, Thompson D, Pronovost PJ (2018). Teamwork in healthcare: key discoveries enabling safer, high-quality care. Am Psychol.

[14] Gao L, Peranson J, Nyhof-Young J, Kapoor E, Rezmovitz J (2019). The role of “improv” in health professional learning: a scoping review. Med Teach.

[15] Hoffmann-Longtin K, Rossing JP, Weinstein E (2018). Twelve tips for using applied improvisation in medical education. Med Teach.

[16] Kaplan-Liss E, Lantz-Gefroh V, Bass E, Killebrew D, Ponzio NM, Savi C (2018). Teaching medical students to communicate with empathy and clarity using improvisation. Acad Med.

[17] Mehta A, Fu B, Chou E, Mitchell S, Fessell D (2021). Improv: transforming physicians and medicine. Med Sci Educ.

[18] Rux S (2020). Utilizing improvisation as a strategy to promote Interprofessional collaboration within healthcare teams. Clin Nurse Spec.

[19] Fu B (2019). Common ground: frameworks for teaching improvisational ability in medical education. Teach Learn Med.

[20] Shahawy S, Watson K, Milad MP (2019). The stretch circle: a preoperative surgical team improvisation exercise. Acad Med.

[21] Lobchuk M, Bell A, Hoplock L, Lemoine J (2021). Interprofessional discharge team communication and empathy in discharge planning activities: a narrative review. J Interprofessional Educ Pract.

[22] Stadick JL (2020). Understanding health care professionals’ attitudes towards working in teams and interprofessional collaborative competencies: a mixed methods analysis. J Interprofessional Educ Pract.

[23] Hammer RR, Rian JD, Gregory JK, Bostwick JM, Birk CB, Chalfant L (2011). Telling the Patient’s story: using theatre training to improve case presentation skills. Med Humanit.

[24] Watson K (2011). Perspective: serious play: teaching medical skills with improvisational theater techniques. Acad Med.

[25] Alan Alda Center for Communicating Science [Internet]. [cited 2022 Sep 27]. Available from: https://www.stonybrook.edu/commcms/alda-center/about/index.php.

[26] MacArthur B, Leavey N, Ng A, Newman T (2020). Abandoning the runaway train: slowing down to draw on lessons learned from health communication training. Theory and best practices in science communication training.

[27] MacArthur BL, Lindenfeld LA, Aurbach E, Bevan B, Newman TP (2020). Bridging science with society: defining pathways for engagement. CCJ.

[28] Preis H, Bojsza E, Lindenfeld L, Gan TJ, Pati S (2022). Process evaluation of a medical improvisation program for healthcare communication training. J. Healthc. Commun..

[29] Preis H, Bojsza E, Lindenfeld L, Pati S (2021). Medical improvisation improves communication skills among healthcare professionals. CCJ.

[30] Bauer MS, Damschroder L, Hagedorn H, Smith J, Kilbourne AM (2015). An introduction to implementation science for the non-specialist. BMC PsycholBMC Psychol.

[31] Curran GM, Bauer M, Mittman B, Pyne JM, Stetler C (2012). Effectiveness-implementation hybrid designs: combining elements of clinical effectiveness and implementation research to enhance public health impact. Med Care.

[32] Conlon C, Timonen V, Elliott-O’Dare C, O’Keeffe S, Foley G (2020). Confused about theoretical sampling? Engaging theoretical sampling in diverse grounded theory studies. Qual Health Res.

[33] Creswell J, Plano CV (2017). Choosing a mixed methods design. Designing and conducting mixed methods research.

[34] Chevalier J, Buckles D. Participatory action research: theory and methods for engaged inquiry. 2nd ed. Routledge; 2019. Available from: https://www.routledge.com/Participatory-Action-Research-Theory-and-Methods-for-Engaged-Inquiry/Chevalier-Buckles/p/book/9781138491328.

[35] Higginbottom G, Liamputtong P. Participatory qualitative research methodologies in health. Sage Publications; 2015. Available from: https://uk.sagepub.com/en-gb/eur/participatory-qualitative-research-methodologies-in-health/book239575.

[36] McIntyre A. Participatory Action Research (2008). 2455 teller road, thousand oaks California 91320.

[37] Kools S, McCarthy M, Durham R, Robrecht L (1996). Dimensional analysis: broadening the conception of grounded theory. Qual Health Res.

[38] Schatzman L, Maines D (1991). Dimensional analysis: notes on an alternative approach to the grounding of theory in qualitative research. Social organization and social process: essays in honor of Anselm Strauss.

[39] Aron A, Aron EN, Smollan D (1992). Inclusion of other in the self scale and the structure of interpersonal closeness. J Pers Soc Psychol.

[40] US Department of Health and Human Services Agency for Healthcare Research and Quality. Surveys on Patient Safety Culture (SOPS) Hospital Survey 2.0. 2021. Available from: https://www.ahrq.gov/sops/surveys/hospital/index.html.

[41] Dalbert C (1996). Uncertainty tolerance scale [database record].

[42] Felsman P, Gunawardena S, Seifert CM (2020). Improv experience promotes divergent thinking, uncertainty tolerance, and affective well-being. Think. Ski. Creat.

[43] Cohen S, Kessler RC, Gordon LU (1997). Measuring stress: a guide for health and social scientists. Measuring stress: a guide for health and social scientists.

[44] Lee EH (2012). Review of the psychometric evidence of the perceived stress scale. Asian Nurs Res (Korean Soc Nurs Sci).

[45] Trockel M, Bohman B, Lesure E, Hamidi MS, Welle D, Roberts L (2018). A brief instrument to assess both burnout and professional fulfillment in physicians: reliability and validity, including correlation with self-reported medical errors, in a sample of resident and practicing physicians. Acad Psychiatry.

[46] Salsman JM, Schalet BD, Park CL, George L, Steger MF, Hahn EA (2020). Assessing meaning & purpose in life: development and validation of an item bank and short forms for the NIH PROMIS®. Qual Life Res.

[47] Glaser BG (1967). Strauss AL.

